# Adrenal Insufficiency in Cirrhosis Patients: Evaluation of 108 Case Series

**DOI:** 10.5005/jp-journals-10018-1237

**Published:** 2017-09-29

**Authors:** Hali Rakici

**Affiliations:** Department of Gastroenterology, Recep Tayyip Erdogan Universitesi, Riza, Turkey

**Keywords:** Adrenal insufficiency, Cirrhosis, Prevalence.

## Abstract

**Aim:**

Adrenal insufficiency (AI) in cirrhosis is an issue that has recently gained momentum. It can be seen in both stable and critically ill (sepsis, septic shock, and gastrointestinal system bleeding) cirrhotic patients. Its prevalence exists in a wide range since standardization of diagnostic methods is lacking. We aimed to scrutinize this issue in a 108 case series.

**Materials and methods:**

We studied the presence of AI and its stage in patients with cirrhosis and its complications by using cross-sectional study. Standard-dose short synacthen test (SD-SST) was performed in 108 patients that had Child C decompensated cirrhosis without critical illness and it was aimed to determine the prevalence of AI based on basal cortisol, peak cortisol, and delta cortisol (basal total cortisol minus peak cortisol after stimulation) levels.

**Results:**

The prevalence of AI in cirrhosis was found to be 25% based on basal cortisol level of <140 nmol/L, 22.2% based on delta cortisol level of <250 nmol/L, and 29.6% based on peak cortisol level of <500 nmol/L.

**Conclusion:**

Prevalence of AI shows variation in decompensated cirrhosis without critical illness depending on different measures used. More definite results can be obtained when more standardized criteria are widely put into use.

**How to cite this article:** Rakici H. Adrenal Insufficiency in Cirrhosis Patients: Evaluation of 108 Case Series. Euroasian J Hepato-Gastroenterol 2017;7(2):150-153.

## INTRODUCTION

Adrenal insufficiency is one of the subjects that has become a hot topic in recent years in patients with liver cirrhosis. Adrenal insufficiency in cirrhosis patients stands for the picture that appears as the complication of cirrhosis in the absence of primary AI just as hepatorenal syndrome. Various definitions have been suggested to describe this picture. The definitions of AI in cirrhosis, critical illness-associated corticosteroid insufficiency, relative AI, and hepatoadrenal syndrome are being used. These pictures can be encountered both in stable cirrhosis and in end-stage cirrhosis. This issue gains more importance in cirrhosis accompanied by terminal events, such as sepsis because of consideration whether the use of corticosteroids in the treatment would be beneficial. It is known that the risk of infection is enhanced in cirrhotic patients. Sepsis is one of the prominent causes of mortality in cirrhosis. Similar pathological processes play a role in both cirrhosis and sepsis. Decreased mean arterial pressure, decreased peripheral vascular resistance, hyperdynamic vascular insufficiency, increased cardiac output, reduced sensitivity to vasopressin, increased level of proinflammatory cytokines, such as interleukin-1 (IL-1), IL-6, and tumor necrosis factor alpha (TNF-) are effective in this process and induce AI. Although the reason for AI is not definite, it is attributed to decreased synthesis of total cholesterol, high-density lipoprotein (HDL) cholesterol, and low-density lipoprotein cholesterol in the liver in cirrhosis and increased level of circulating endotoxins, such as proinflammatory cytokines and lipopolysaccharides.^1-8^

Cortisol is secreted from adrenal cortex and controlled by adrenocorticotropic hormone (ACTH), which is released from the pituitary gland. Secretion of ACTH is controlled by corticotropin-releasing hormone, which is released from paraventricular nucleus of the hypothalamus. Cortisol is not stored in the body. Stress plays an important role in the synthesis and production of Cortisol. Hypothalamo-pituitary-adrenal (HPA) axis is activated by stress and serious diseases. Appropriate amount of Cortisol is required to inCrease vasCular tone and cardiac output. More than 90% of circulating cortisol is bound to corticosteroid-binding globulin (CBG) and albumin (5%). Approximately 10% is found as free cortisol. Cortisol is turned into inactive metabolite cortisone by 11-beta hydroxysteroid dehydrogenase type I in the liver. Free cortisol may increase in cirrhosis due to hypoalbuminemia. It may also increase in sepsis and critical illness as the level of CBG is decreased. Reduced cholesterol level in cirrhosis leads to low production of cortisol in particular, which is synthesized from HDL cholesterol. In addition to increase in vascular tone and cardiac output, cortisol also enhances protein catabolism and reduces lipolysis, hyperglycemia, and cytokine production.^9-16^

Adrenal insufficiency in cirrhosis is encountered not only with stable and critical illness, but also during early and late phases of liver transplantation. Its prevalence may exist in a wide range depending on the method used. Various studies reported its prevalence in a quite wide range as 10 to 87% in critically ill cirrhotic patients, 7 to 83% in stable cirrhotic patients, and 61 to 92% in patients with liver transplantation. Different rates found in different studies might have resulted from differences in the diagnostic methods used and different standards determined for these methods.^16-21^

Clinical diagnosis of AI in cirrhosis is difficult. There are no given symptoms. According to the Endocrine Society clinical practice guidelines, essential criteria for diagnosis of primer AI is that the morning basal cortisol level, ACTH level, and peak cortisol level be lower than 500 nmol/L.^5^

Diagnostic methods include serum total cortisol level, SD-SST, low-dose SST (LD-SST), peak cortisol level, and free cortisol level. Delta cortisol and salivary cortisol tests are recommended by some authors.^1-4,18-22^ Although insulin-induced hypoglycemia test is the method that demonstrates HPA axis best, it is not appropriate to be used in cirrhotic patients because it can cause serious problems in serious cases. Since each of these tests is taken as a measure in various studies and the values are different from each other, prevalence rates are determined in a wide range. For example, there are different studies that took the cut-off value for basal plasma cortisol as <280, <250, or <138 nmol/L. In SD-SST, serum total cortisol level is measured at the 30th and 60th minute after 250 jug synacthen is administered via IV or intramuscular route. A peak cortisol level <552, <500, or < 442 nmol/L, or a delta cortisol level <250 nmol/L is taken as the measure. In LD-SST test, serum total cortisol level is measured at the 30th minute after 1 ug synacthen is administered via IV route.

Although free cortisol level is a better diagnostic method, it is not used in daily practice. Salivary cortisol is used, but it is influenced by oral hygiene and bleeding, which reduce its diagnostic value.^17,22-25^

Although corticosteroid therapy in AI in cirrhosis has been reported to be either beneficial or nonbeneficial in various publications, it is still debatable. Nevertheless, it may be beneficial in some cirrhotic patients with vasopressin-resistant shock.^26-29^

## MATERIALS AND METHODS

A total of 108 patients with decompensated cirrhosis (Child C), who were hospitalized at the Training and Research Hospital, Gastroenterology Clinic, between January 2013 and December 2015 due to cirrhosis and complications (gastrointestinal hemorrhage, hepatic encephalopathy, tense ascites, spontaneous bacterial peritonitis, hyponatremia, and hepatorenal syndrome), were enrolled in the study. We studied the presence of AI and its stage in patients with cirrhosis and its complications by using cross-sectional study. All patients were hemodynamically stable (without critical illness). Approval of the Ethics Committee was obtained. The patients were prospectively evaluated. Basal cortisol and ACTH levels were studied in the morning between 8 and 8.30 a.m. Cortisol level was studied at the 30th and 60th minute using SD-SST. Peak cortisol and delta cortisol (peak cortisol minus basal cortisol) levels were studied. In SD-SST test, cortisol level was studied at the 30th and 60th minute after 250 ug tetracosactide acetate (Synacthen; Novartis Pharma AG, Basel, Switzerland), which is a synthetic ACTH, was administered via IV route. Patients with normal ACTH level were enrolled in the study to exclude primary AI. It was aimed to evaluate AI in decompensated cirrhotic patients without critical illness.

Categorical variables were compared by Pearson chi-square test and Fisher’s exact test. Level of alpha (p) significance was considered to be 0.05. Analyses were done using Statistical Package for the Social Sciences version 19.0 statistical package program.

## RESULTS

Patients consisted of 48 females and 60 males. The youngest one was 31 years and the oldest was 82 years. With regard to the etiological diagnoses of the patients, it was determined that cirrhosis resulted from chronic hepatitis B in 36 (33.3%), hepatitis C in 33 (30.6%), nonalcoholic steatohepatitis (NASH) in 18 (16.7%), cryptogenic cirrhosis in 12 (11.1%), alcoholic hepatitis in 6 (5.5%), and congenital hepatic fibrosis in 3 (2.8%) patients. All patients with decompensated cirrhosis were hemodynamically stable at the time of testing.

With regard to MELD (Model for End-Stage Liver Disease) score, it was >14 in 63 (58.3%) patients and <14 in 45 (41.7%) patients. Ascites was present in all patients. History of gastrointestinal bleeding was present in 66 (61.1%) patients; 57 (52.8%) patients had encephalopathy, 18 (16.7%) patients had spontaneous bacterial peritonitis, and 15 (13.9%) patients had hyponatremia. Prevalence of AI was 25% (27/108) when a basal cortisol level <140 nmol/L was taken as the basis, 22.2% (24/108) when a delta cortisol level <250 nmol/L was taken as the basis, and 29.6% (32/108) when a peak cortisol level <500 nmol/L was taken as the basis. When basal cortisol level <140 nmol/L was taken as the basis, prevalence of AI was 38% (24/63) in the patients with MELD score >14 and 6.7% (3/45) in the patients with MELD score <14. When delta cortisol level was taken as the basis, prevalence of AI was 19% (12/63) in the patients with MELD score >14 and 33.3% (15/45) in the patients with MELD score <14. When peak cortisol level was taken as the basis, prevalence of AI was 30% (21/63) in the patients with MELD score >14 and 24.4% (11/45) in the patients with MELD score <14. Although prevalence of AI was found to be close to the statistical significance (p = 0.051) in the patients with MELD score >14 *vs* <14 when basal cortisol level was taken as the basis, no significant difference was determined when delta and peak cortisol levels were taken as the basis. The results are demonstrated in Tables 1 and 2.

**Table 1:** **Table 1:** Prevalence of AI

		*Prevalence of AI*			
*Methods*		+		–	
Basal cortisol level <140 nmol/L		27 (25%)		81 (75%)	
Peak cortisol level <500 nmol/L		32 (29.6%)		76 (70.4%)	
Delta cortisol level <250 nmol/L		24 (22.2%)		84 (77.8%)	

**Table 2:** **Table 2:** MELD score and prevalence of AI

*MELD score*		*Basal Cortisol level <140 nmol/L*		*Peak cortisol level <500 nmol/L*		*Delta cortisol level <250 nmol/L*	
>14		24/63 (38%)		21/63 (30%)		12/63 (19%)	
<14		3/45 (6.7%)		11/45 (24.4%)		15/45 (33.3%)	

## DISCUSSION

Adrenal insufficiency in cirrhosis or, in other words, hepatorenal syndrome is an issue that has recently gained momentum. However, lack of definite standards defined for diagnostic methods and, on the contrary, indefinite physiopathology of the disease make this issue debatable. Three reviews that have been published recently^1-3^ held this issue in detail and mentioned about difficulties in diagnosis and treatment. Adrenal insufficiency in cirrhosis can be seen both in stable cirrhosis and in cirrhosis with critical illness (sepsis, septic shock, variceal bleeding). Although its incidence is in line with the severity of cirrhosis, it exists in a wide range depending on the method used.^11-14,21,24,25^ The incidence is higher when AI is determined based on basal cortisol, SD-SST, and delta cortisol (basal total cortisol minus peak cortisol after stimulation), whereas it is found to be lower when AI is determined based on LD-SST, free cortisol, and salivary cortisol. However, the latter three tests have limited use in daily practice. Insulin-induced hypoglycemia, which reflects HPA axis best, cannot be performed in many of these patients. In the present study, all cases were stable cirrhotic patients. Adrenal insufficiency was investigated in stable or, in other words, noncritically ill cirrhotic patients and the incidence was found to range between 7 and 83% depending on the method used. Level of hypoalbuminemia and CBG decreases as the severity of cirrhosis is increased; hence, this leads to overdiagnosis when total cortisol level is taken as the basis. In the present study, prevalence of AI was 25% when total cortisol level was taken as the basis, 22.2% when delta cortisol level was taken as the basis, and 29.6% when peak cortisol level was taken as the basis. In this study, CBG level is not examined and this should be recorded as a limitation. With regard to the prevalence of AI based on a MELD score >14 or <14, the prevalence of AI is 6.7 *vs* 38% according to the basal cortisol level in those with a MELD score >14 was considered to be close to the statistical significance (p = 0.051). No significant difference was determined when delta and peak cortisol levels were taken as the basis. Detection of free cortisol is not used in routine practice since it is both difficult and expensive. Free cortisol can be calculated over serum total cortisol and CBG levels using Coolens’ formula.

Although the reason for AI is not definite, some hypotheses have been suggested. These include low HDL and total cholesterol levels, increased proinflammatory cytokines (IL-1, IL-6, TNF-alpha), endotoxemia (lipopolysaccharide), decreased apolipoprotein A-1 level, and hemorrhage and infarction in the adrenal gland due to proclivity to hemorrhage and thrombosis in cirrhosis. All these factors may lead to HPA dysfunction. Moreover, translocation of enteric bacteria as an inflammation-enhancing factor may cause glucocorticoid resistance.^15^

Effect of corticosteroid therapy in AI in cirrhosis is debatable. In addition to the papers reporting that it is beneficial, there are papers reporting that it is not. However, it can be used in vasopressor-resistant shock. It is reported that corticosteroid therapy reduces need for vasopressor in critical illness, shortens staying at intensive care unit, enhances resolution of shock, but increases proclivity to infection and bleeding.^1-4,26-29^

Since all cases in the present study were stable cirrhotic patients, we were unable to assess the effect of corticosteroid therapy.

In conclusion, steroid therapy is not necessary in non-critically ill cirrhotic patients since they are usually hemo-dynamically stable. The prevalence of AI would be exposed more definitely when free cortisol and salivary cortisol are widely put into practice and standardized better.
